# Status of the burbot (*Lota lota* L.) in the Lower Danube (Bulgaria) – a species threatened by climate change

**DOI:** 10.3897/zookeys.910.47866

**Published:** 2020-02-10

**Authors:** Pencho G. Pandakov, Teodora M. Teofilova, Nikolay D. Kodzhabashev

**Affiliations:** 1 Forestry University, Faculty of Forestry, Department of Hunting and Game Management, 10 Kliment Ohridski Blvd., 1756 Sofia, Bulgaria Forestry University Sofia Bulgaria; 2 Institute of Biodiversity and Ecosystem Research (IBER), Bulgarian Academy of Sciences, 1 Tsar Osvoboditel Blvd., 1000 Sofia, Bulgaria Institute of Biodiversity and Ecosystem Research, Bulgarian Academy of Sciences Sofia Bulgaria

**Keywords:** Age structure, conservation status, fecundity, fragmentation, growth, length-weight relationship

## Abstract

The study provides data on the catch composition, length-weight relationship, age structure, gender structure, growth, maturation, fecundity, distribution and conservation status of the burbot *Lota lota* (Linnaeus, 1758) in Bulgaria. During six consecutive winters (2008–2014) a total of 395 burbot specimens were caught. The total length and the weight of the specimens ranged from 16 to 51 cm and 29.8 to 1057 g, respectively. Seven age classes were represented (3- to 9-years-old), with 3-, 4-, and 5-years-old most abundant. The maximal life expectancy was estimated as 12 years. Male-female ratio was 1:1. Maturity happens at the age of four at the earliest, valid for both sexes. One-quarter of the fish, older than 5 years were determined as non-reproducing in the particular year. The absolute fecundity varied between 47 462 and 810 236 eggs for females ranging from 5 to 7 years old and from 25.7 to 41.5 cm in length. A dramatic decrease of burbot population was observed in the last two decades. Warming water temperatures of the Danube, together with fragmentation in its tributaries are considered as the major threats affecting the species. Therefore, the burbot in Bulgaria is classified as Endangered.

## Introduction

The burbot is the only freshwater gadoid fish species (Berg 1949). It is thought this species’ thermal preference is ecologically intermediate between thermal preferences of cold-water salmonids and more thermophilic cyprinids (Nikcevic et al. 1995). Burbot usually reduces and even arrests its feeding at summer temperatures, falling into a state of hibernation (Tyulpanov 1967; Hardewig et al. 2004). The species’ behavior can be defined as crepuscular and nocturnal.

Although burbot is abundant throughout much of its natural range, there are many populations that are extirpated, endangered, or in serious decline (Stapanian et al. 2010). Burbot is thought to be an excellent indicator for habitat degradation and an early indicator of climate change (Stapanian et al. 2010; Edwards et al. 2011). In Europe, burbot have been extirpated from the United Kingdom, Belgium, and parts of Germany as result of the deterioration of habitats (Stapanian et al. 2010). In the Middle and Upper Danube basins, the species is considered vulnerable (Stapanian et al. 2010). The reported declines in the burbot populations in northern Europe is caused by pollution, acidification, eutrophication, fragmentation and invasive species (Pulliainen and Korhonen 1993; Kjellman 2003; Stapanian et al. 2010). Damming, river regulation, and pollution have caused the extirpation and decline of the species in Poland, Slovakia, Czech Republic, and Slovenia (Brylińska et al. 2002; Slavík and Bartoš 2002; Holcík 2003; Kruk 2007; Stapanian et al. 2010).

**Figure 1.**
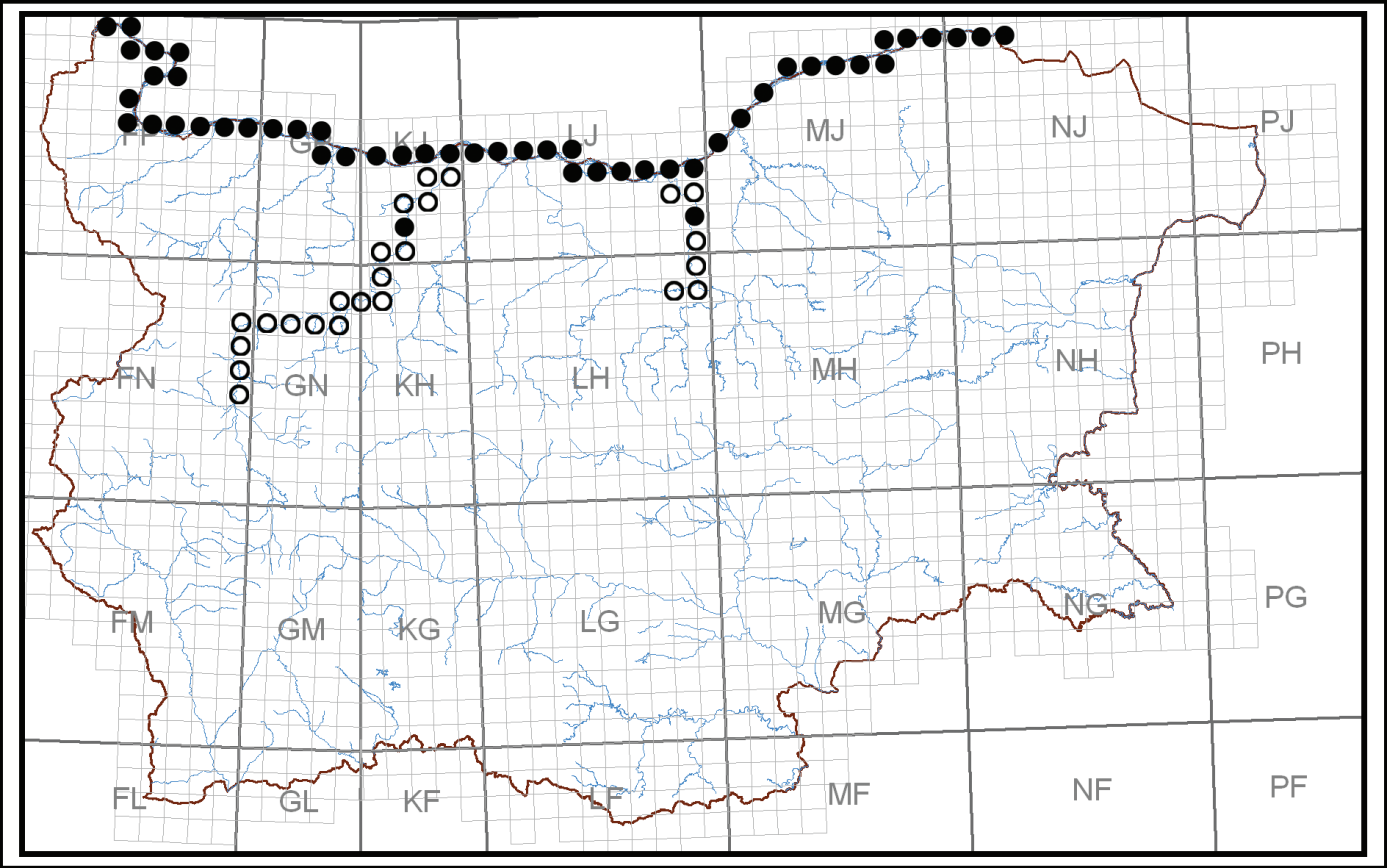
Distribution of the burbot in Bulgaria presented via registration of the species in UTM 10 × 10 km grid (○ localities before 2002, ● localities after 2002).

The species was widespread and very numerous in the Bulgarian section of the Danube during the first half of 20^th^ century (Kovachev 1923; Drensky 1951). Later, Pomakov (1985) reported a dramatic decline over 30 years and classified the species as Endangered EN, mentioning that it had not been found since 1969. Burbot has since been down-listed to Vulnerable VU by Trichkova and Stefanov (2011) using data from the end of the last and the beginning of this century when the commercial burbot catch in the Danube had increased significantly. Karapetkova and Zhivkov (2010) have classified the species as occasional and threatened by extinction. The species has lost most of its localities in the major Danube tributaries in Bulgaria (Fig. 1).

Burbot also represents a target species for some commercial fisheries during winter, especially for the eastern Bulgarian section of the Danube. Listed as a game fish, size and harvest limits are imposed by the Fisheries and Aquaculture Act in Bulgaria. A few studies of populations from the Serbian part of the Middle Danube have been published during the last decades (Janković 1986, Nikcevic et al. 1995; Skorić et al. 2013; Višnjić-Jeftić et al. 2014; Smederevac-Lalić et al. 2015). Our study represents the first scientific survey of burbot populations in the Lower Danube and in particular in Bulgaria, since the species had been historically recorded by Kovachev (1923). We aim to provide biological data on the burbot population in the Lower Danube, to clarify the national Bulgarian conservation status of the species, and to provide data for the revision of its international conservation status. Currently, the species is listed as Least Concern LC in International Union for the Conservation of Nature IUCN Red List, and although the population trend and major threats are mentioned as unknown, there are a number of publications reporting severe negative impacts affecting the species worldwide (Stapanian et al. 2010).

## Material and methods

Field sampling was carried out from 2008 to 2014 in the Bulgarian section of the Danube River between the village of Malak Preslavets (km 412 on the river) and the town of Silistra (km 375). This short section of the river (only 37 km) was selected as a model case, because the commercial fishing efforts targeting the species are restricted here. More than 96% of all registered catches are coming from this stretch. In the rest of the Bulgarian section of the Danube, burbot catches are accidental. The provided fish for the study were caught by commercial fishermen, using hoop nets deployed at night or twilight in December and January along the shoreline in shallow waters with sandy-gravelly substrate and low to moderate flow velocity.

Collected fishes were immediately chilled in ice and transferred to the laboratory. All individuals were measured without any fixation and total length TL and wet weight (W) were determined. The length-weight relation was expressed using the equation W = aTL^b^ (Ricker 1975), where *a* is the intercept and *b* is the slope of the log-transformed relation (Froese 2006). The parameters *a* and *b* were estimated by linear regression from the logarithmically transformed equation: log10W = log10a + blog10TL. Normal distribution of the data sets was checked, an F-test was performed to determine if the variances of the data sets were equal, and a t-test was applied to test the isometric pattern hypothesis (b = 3) and the significance in the difference between the mean length and weight of examined males and females by age classes. For all statistical analyses we rejected the null hypothesis if the *p*-value was less than 0.05.

We used vertebrae to estimate the age of the fishes, a method verified for the burbot by Guinn and Hallberg (1990). The individual vertebrae, taken from the zone between third and seventh column vertebrae, were freed from the connective tissue and cartilage of the vertebral column, without previous boiling or chemical immersion, using a scalpel and clamps, and then air dried. The age of the fish was rounded to the next integer number.

The gonads were removed from all individuals and the sex was determined. Specimens without any formed reproductive glands were perceived as juveniles. Those whose glands were thread-like were perceived as immature and a histological examination was used for their sex determination. Individuals with well-developed gonads, not containing sexual products together with the immature ones were grouped and classified as non-reproducing. The absolute fecundity of all 10 matured females in one of the collected catches (from 25.12.2008) was estimated. Their ovaries were weighed, and the eggs in the ovary were subsampled, counted using a stereomicroscope, weighed, and the total number calculated by direct proportion.

Growth rate of the population was described via the Bertalanffy Growth Function (Bertalanffy 1951) using Chapman’s method (Chapman 1961) for the estimation of the growth parameters from observed length-age data: Lt = L∞ (1 – exp (–k (t – t_0_))), where Lt is the total length at age t, L∞ is the length that the fish would reach at an infinitely high age or the asymptotic length at which growth is zero, and k is the curvature parameter; and t_0_ is the theoretical age when the fish has zero length.

The statistical calculations as well as all figures and tables were performed using MICROSOFT OFFICE 2010. A distribution map was created using ADOBE PHOTOSHOP 7.0.

The conservation status of the species was classified according IUCN criteria (IUCN 2012b), applied at regional level (IUCN 2012a). Official data submitted by the Executive Agency of Fisheries and Aquaculture EAFA and Executive agency for exploration and maintenance of the Danube river EAEMDR, supplemented by ours, were used for that purpose.

## Results

### Catch composition by size and seasons

A total of 395 burbot specimens were obtained. All of them were collected in first three seasons of the survey: 97 specimens in 2008/2009, seven specimens in 2009/2010, and 291 specimens in 2010/2011 winter seasons. In the winter of 2011/2012 all caught burbot were immediately released by the fishermen because of their small size (under the limit in the regulatory framework). During the winter 2012/2013 and 2013/2014 seasons the catches were extremely poor so we had no fish for examination.

**Figure 2.**
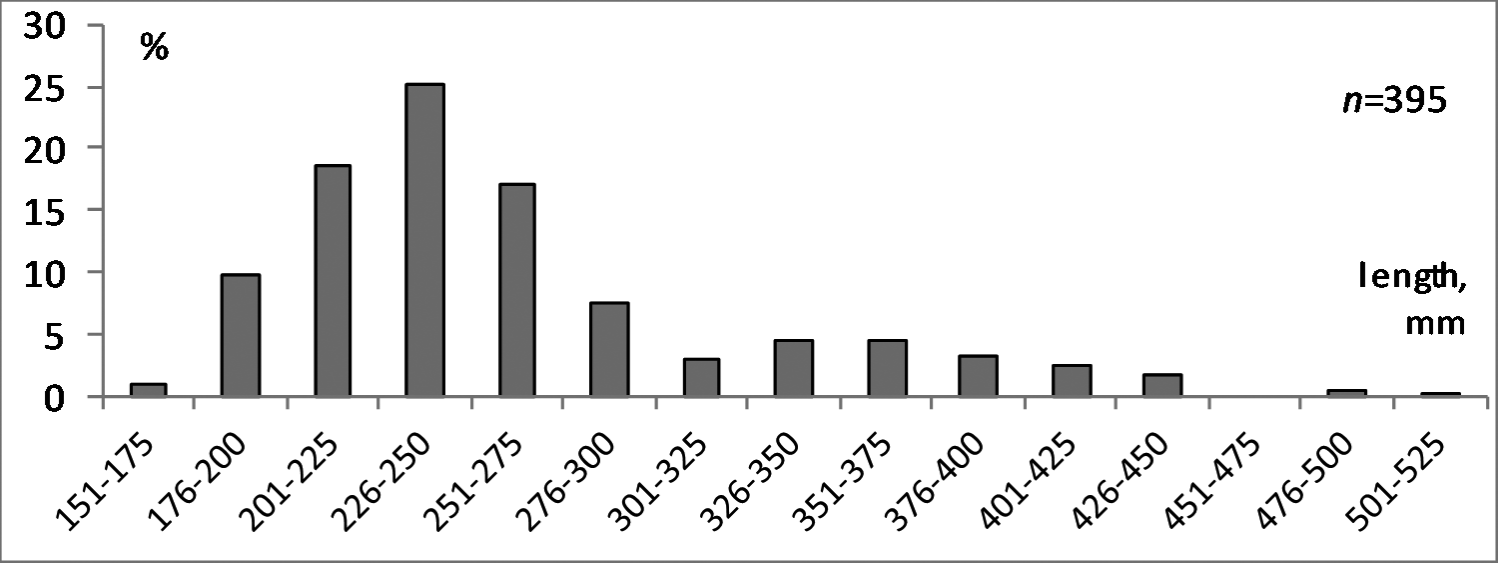
Distribution (in %) of body length among studied burbots, as represented by 25-mm size classes.

Total length of the fish varied within the range of 160–510 mm (average: 263 mm) and body weight from 29.8 to 1057 g (average: 160 g). Length groups were established at 25 mm and almost 80% of caught individuals referred to the first five size classes and only 20% were distributed among the other eight classes (Fig. 2).

### Length-weight relationship

**Figure 3.**
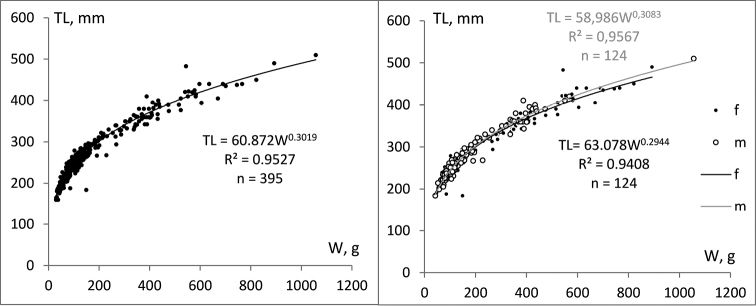
Length-weight relationship for all collected fish (left), and separate relationships for the both sexes (right); TL = total length and W = weight.

The length-weight relation was calculated for all 395 individuals regardless of their gender (Fig. 3, left). The data were well described by the gradient curve expressed by the next equation: TL = 60.872W^0.3019^. There were not significant differences in mean length and weight among females and males for 3-, 4-, and 5-year-old fish (*t*-test, *p* > 0.05). Significant difference in mean length and weight among females and males (*p* = 0.007) was found for 6-year-old females, which were characterized by a stronger decrease in length growth than the 6-year-old males. Different sexes in the other three age classes (7-, 8-, and 9-year-old fish) were not numerous enough for implementing such analysis. However, we described the length-weight relation for both sexes separately (Fig. 3, right), although significant difference among males and females was not found without taking in to account the age.

**Figure 4.**
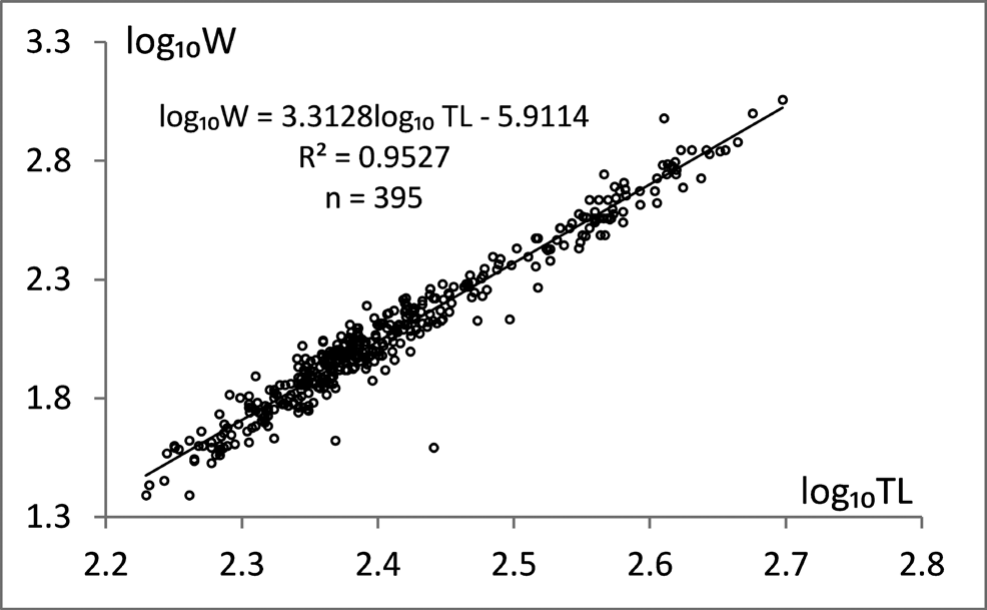
Length-weight relationship for the total catch expressed by linear equation.

The parameters *a* and *b* were estimated by linear regression (Fig. 4) of the logarithmically transformed equation: log10W = 3.3128log10TL – 5.9114, with coefficient of determination (R²) of 0.9527. The value of the parameter b = 3.3128 was significantly different from 3, showing positive allometric growth.

### Age

**Figure 5.**
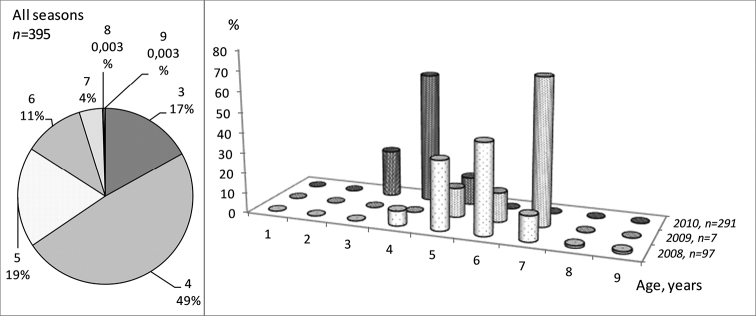
Age composition (in %) of all collected burbot (left) and for each season separate (right). On the left diagram, the upper value is the age category and lower value is the percentage of this category in total number of fish.

All caught burbot exhibited an external incomplete year ring referring to the unfinished annual cycle. Most of the fish had the first inner hyaline circle on the base of the vertebra hardly visible (or sometimes absent), which corresponds to the very short (or sometimes absent) inactive period during the first summer. Age composition of the catches during the whole period, and for each season separately, is visualized in Figure 5. Most fish were 3, 4, and 5 years old. Seven different ages were represented in the catch, from 3- to 9-year-old fish.

The abundance of the age groups was the highest during the first winter season. The second season was characterized with only few obtained specimens, but all individuals were in a reproductive age distributed over only three age groups. During the winter of 2010/2011 (third season) the quantity of the caught fish was the highest but only composed of three age classes, and mainly by young individuals (3- and 4-year-olds).

### Gender and maturation

With regards to sex composition, male-female ratio was 1:1 (124 males, 124 females). Males were slightly more numerous in the younger ages, while the females dominated in the elder ones. Our study showed that burbot in Bulgaria reach sexual maturity for the first time during their fourth year. The group of non-reproducing fish constituted 34% of the fish older than four years and 25% of those older than five. In our catch, all 3-year-old burbot were juvenile or immature. At age of four, about two-thirds of the specimens are still immature in both sexes. Five-year-old and older specimens were generally mature (Table 1).

**Table 1.** Sex composition of the captured burbot.

Age	Males	Females	Juveniles
**3**	7	7	53
**4**	62	47	83
**5**	29	33	11
**6**	24	20	0
**7**	1	16	0
**8**	0	1	0
**9**	1	0	0
**Total**	**124**	**124**	**147**

Although the fish were caught in the spawning season, there were females and males whose gonads were thin, small, and without any products. The gonads were unlike an empty sack, so it can be excluded that these fish had already released their eggs or semen before they were caught. However, most of the fish older than five years had well-developed gonads in the last stage of maturity.

### Fecundity

The fecundity varied between 47 462 and 810 236 eggs for females ranging in age from 5 to 7 and from 257 to 415 mm in length. The fecundity of 5-year-olds was about 10 times less than 7-year-olds. There were positive correlations between the number of the eggs and the age of the females (r = 0.74), as well as between the number of eggs and the total length (r = 0.87) and the weight (r = 0.97) of female burbot.

### Growth

**Figure 6.**
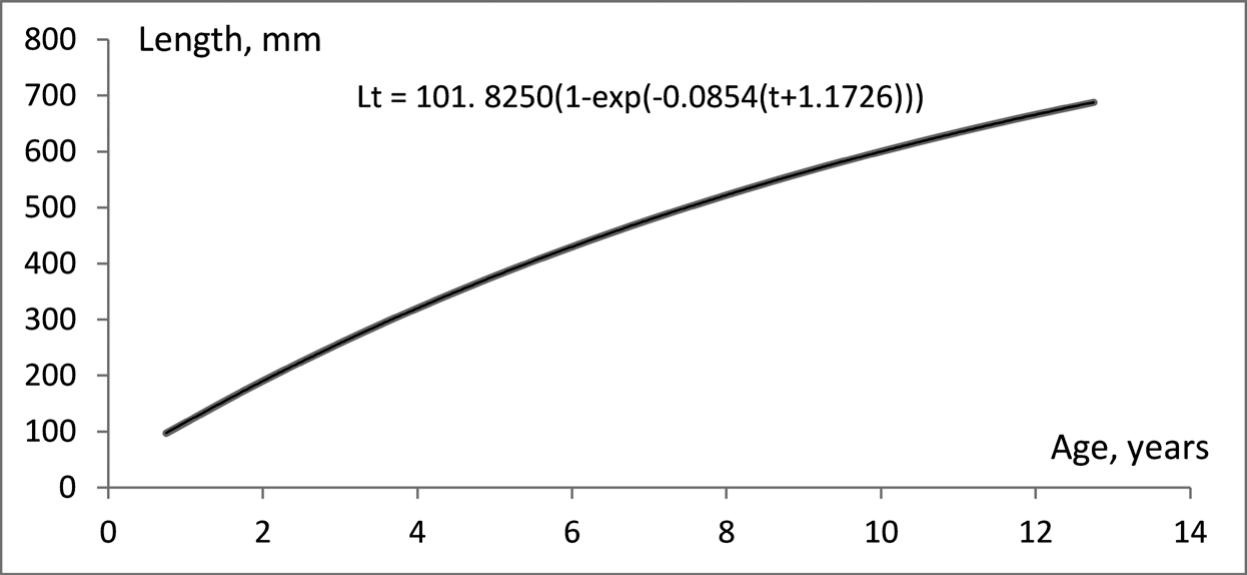
Growth curve of burbot in Bulgaria.

The values of the parameters *K*, *L*∞ and *t_0_* in the von Bertalanffy growth equation were established as follows: K = 0.085426, L∞ = 1018.25, t_0_ = −1.17262. The growth rate can be described by the function: Lt = 101.8250(1 – exp (−0.0854(t + 1.1726))) (Fig. 6). Based on the parameters of the established growth curve, young fish (up to 2 years) increase their size quickly, while later length growth gradually decreases (Table 1). According to the local fishermen, the largest captured specimens were always males with an approximate weight of 2.2 kg. Using the polynomial equation established for males in Figure 3 (right), we calculated that these largest fish should be approximately 633 mm long, which equates to a maximum lifespan of the burbot in Bulgaria of about 12 years (Table 2).

**Table 2.** Simulation of age-length relationship based on the von Bertalanffy equation.

Age		Total length, mm
1	1^st^ winter	97
2	2^nd^ winter	172
3	3^rd^ winter	242
4	4^th^ winter	305
5	5^th^ winter	364
6	6^th^ winter	417
7	7^th^ winter	466
8	8^th^ winter	512
9	9^th^ winter	553
10	10^th^ winter	591
11	11^th^ winter	626
12	12^th^ winter	658

## Distribution and conservation status

**Figure 7.**
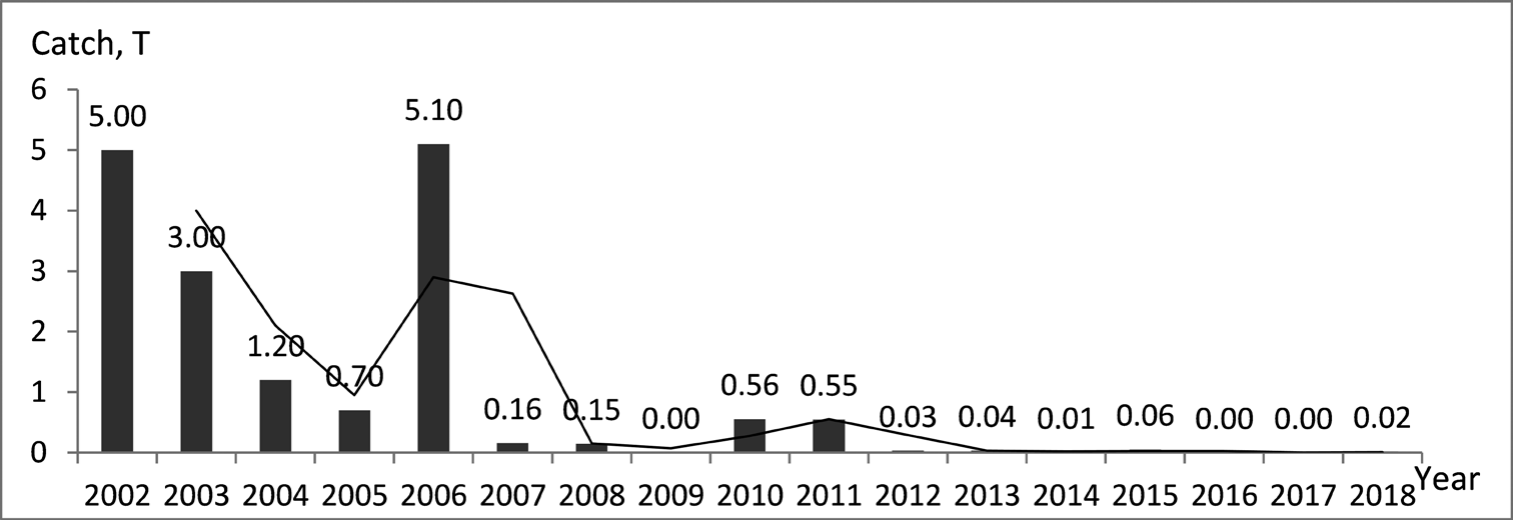
Commercial catch of burbot in Bulgaria, represented by calendar years (data source: The Executive Agency of Fisheries and Aquaculture (EAFA)).

**Figure 8.**
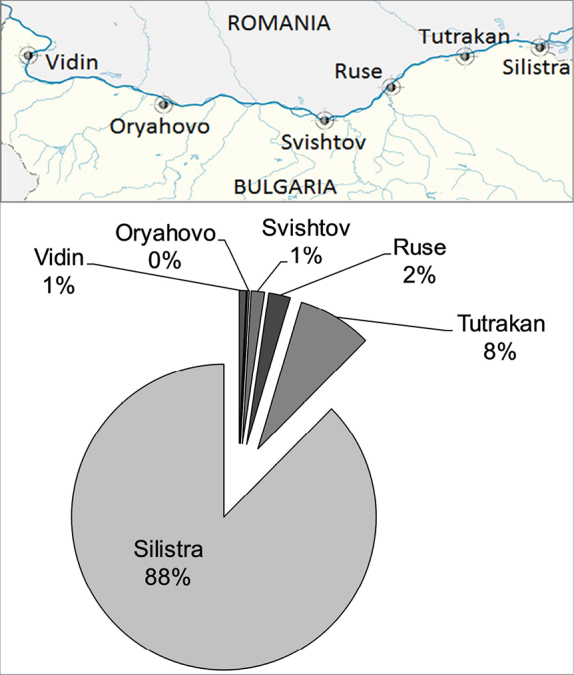
Map of Bulgarian section of Danube (above) and distribution of the catches by place of landing for 2006–2018 (below) (data source: The Executive Agency of Fisheries and Aquaculture (EAFA)).

There was a strong decline (>90%) in the registered catches by EAFA over the period from 2002 to 2018 (equivalent to 3–4 burbot generations) (Fig. 7). About 96% of the commercial catch comes from the eastern river section between Tutrakan and Silistra (Fig. 8) where the fishing effort targeting at this species is concentrated. The negative trend was shown clearly via the moving average trend line (Period = 2), which smooth out the yearly fluctuations. Therefore, the conservation status of the burbot in Bulgaria was classified as Critically Endangered (CR A2cd) according to IUCN criteria (IUCN 2012b). The category was down-listed in Endangered (EN°), after correction for regional level of assessment (IUCN 2012a) due to rescue effect of Romanian and Serbian populations in the Danube and because of historical fluctuations in the commercial catch (Drensky 1951; Pomakov 1985; Trichkova and Stefanov 2011). The past and the present distribution of the species in Bulgaria is shown on Figure 1.

## Discussion

The positive allometric growth (b > 3) shown by our data means that the fish become less elongated or more roundish as they grow. In contrast, a negative allometric growth pattern was reported by Smederevac-Lalić et al. (2015) for the burbot population at Bachka Palanka (middle part of the Danube), although the fish had been caught in the same season. We assumed these differences in growth patterns may be attributed to different gonad development and food availability, as these are primary factors influencing body shape (Jennings et al. 2001). In our study, smaller fish had empty stomachs and larger ones exhibited in most cases developed gonads and prey in the stomach, which contributed to the positive allometric growth curve. We assume that the length growth of reproductive age females slows down faster as compared to the reproductive age males. However, this could not be proven statistically for the fish older than six years due to only few individuals at this age. This corresponds with the conclusion of Žiliukiene and Žiliukas (2010) that differences in growth rates of fish species are more pronounced in the older age groups.

The hardly visible first inner hyaline circle does not mark the end of the first biological year. It is formed during the comparatively short summer inactive period in the first year. This period is shorter in juvenile burbot than in older ones, as juveniles are more tolerant to higher temperatures (Nikcevic et al. 1995; Wolnicki et al. 2002), less stored glycogen and fat in the liver, and they stay active almost throughout the first year of their life in order to increase their size rapidly (Ryder and Pesendorfer 1992; McPhail and Paragamian 2000). Ryder and Pesendorfer (1992) found that larval growth is rapid in May and June but slows down in August, which represents only a short period of limited activity. The use of vertebrae for age determination in burbot proved to be very easy and reliable. As a disadvantage, it requires killing of the fish to obtain the aging structure. Specimens with extracted vertebrae also cannot easily be sold on the fish market, but this is also the case with the other commonly used methods based on otoliths (Guinn and Hallberg 1990; Pulliainen and Korhonen 1990).

The established sex ratio was similar as the reported by Pulliainen and Korhonen (1990) and Smederevac-Lalić et al. (2015). The domination of females in older ages may correspond to differences in the lifespan between sexes, but this remains speculative. Most authors agree that males mature about a year earlier than females (Janković 1986; Arndt and Hutchinson 2000; McPhail and Paragamian 2000), but this was not confirmed by our data. Probably some of the non-reproducing 5-year-old individuals were still immature but this is hardly relevant to the 6-year-old fish. Their sex was clearly evident, and we could not exclude the possibility if their maturity was delayed or if they were non-reproducing or even sterile. Many authors had reported high percentage of non-reproducing fish for adult burbot (Hewson 1955; Evenson 1990; Pulliainen and Korhonen 1990, 1993). Segerstråle (1945) and Sandlund et al. (1985) suggested that burbot may have one or more rest years to restore their nutrition reserves after spawning. Baggerman (1980) and Stacey (1984) point out that exogenous factors, such as water temperature, salinity, photoperiod, and also social factors can delay sexual maturation. Instead, Pulliainen and Korhonen (1990, 1993) connected the large number of non-reproducing burbot with contaminants in the water, which cause sterility, than to presence of rest years.

The observed values of absolute fecundity were very similar to those reported by Brylińska et al. (2002) from Lake Hańcza, Poland. Similar size-egg number correlations can be found in other burbot populations, while the number of the eggs vary from 6300 (Miller 1970) to 3 477 699 (Roach and Evenson 1993).

Comparable growth and life expectancy (maximum ~12 years) of burbot in Southeast Europe were reported by Simonović (2001), Višnjić-Jeftić et al. (2014), and Smederevac-Lalić et al. (2015), but in cold water habitat such as the glacial lake Plav (Montenegro) it can reach a larger body size, up to 6 kg (Goran Tokić pers. comm.). According to McPhail and Paragamian (2000), the lifespan of the southern populations is shorter than in northern ones. Magnin and Fradette (1977) noted that individuals older than 7 years are rare in a population at 45°N, but at 55°N most adults are 8- to 12-years-old. The maximum ages recorded in northern populations were about 20–22 years (Guinn and Hallberg 1990). The growth rate of burbot in Romania, as reported by (Oţel 2007), is very similar to our results (Table 1).

**Figure 9.**
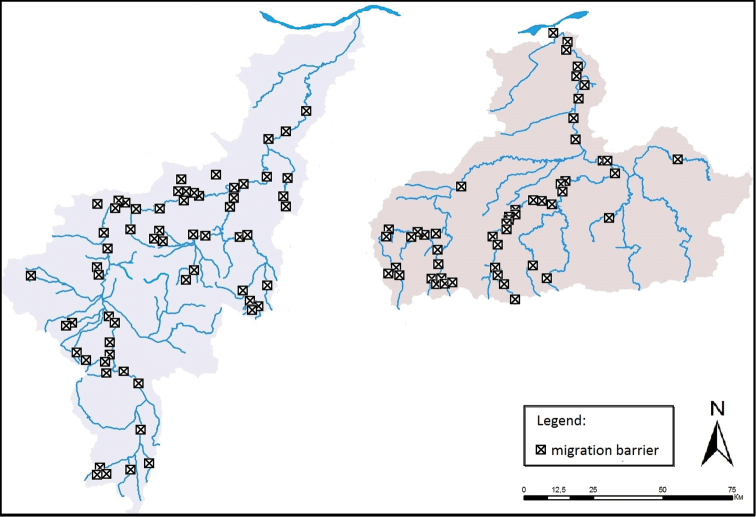
Migration barriers along Iskar (left) and Yantra (right) river catchments (data source: Basin Directorate Danube Pleven 2016).

Data clearly show that burbot is declining at least since the beginning of the century. Chronologically, the species was classified widespread and very numerous (Drensky 1951), Endangered (Pomakov 1985), occasional and threatened by extinction (Karapetkova and Zhivkov 2010), and Vulnerable (Trichkova and Stefanov 2011). In the past this species had been found in the Danube and in the lowland and upland sections of the Iskar and Yantra rivers, its largest tributaries (Kovachev 1923; Morov 1931; Shishkov 1939; Drensky 1951). At present, the species has lost most of its habitats in the tributary rivers, as they have been heavily affected by water pollution during 1960s to 1980s (Rusev 1994), as well as by fragmentation, which is a strong negative impact on this migratory species (Fig. 9). Adult burbot tend to be middle-distance migrants (Waidbacher and Haidvogl 1998) and travel several to more than 250 km in one direction during seasonal migrations (Evenson 1993; McPhail and Paragamian 2000).

Nowadays, burbot inhabits primarily the Danube River and occasionally the Iskar (two adult specimens at Pisarovo village in 2009 during spring high waters; unpubl. data) and Yantra (one adult near the town of Byala in the summer of 2006 (Mihov and Koev 2006), and one adult at the same place in the summer of 2010 (unpubl. data)). The species has been recorded also in Skat River (Ogosta river basin), very close to its mouth near the Danube (Stefanov pers. comm.) (Fig. 1).

Stapanian et al. (2010) noted that the lack of trend data for many burbot populations has left them vulnerable to overfishing. Moreover, burbot is very susceptible to overfishing because much of the fishing effort typically targets aggregations during spawning and feeding (Ahrens and Korman 2002). On the other hand, fishing efforts in Bulgaria are mainly concentrated in the eastern section of the Danube from Tutrakan to Silistra, which represents only part of the potential habitat of the species.

As the burbot`s life cycle is cold-water dependent, Bulgarian populations live at the limit of the species’ preferences, since these populations are at the southern edge of the burbot’s range (Pomakov 1985). Therefore, the Bulgarian populations are especially vulnerable to habitat changes caused by climate change. For example, increases of water temperatures due to the climate change have been noted as a reason for declines in Lake Oneida (New York, USA) at the southern extent of the species’ range (Jackson et al. 2008). According to the local Bulgarian fishermen, several years with abundant burbot are followed by a longer period of poor catches. Similar fluctuations were reported by (Švagždys 2002), who concluded that a warming climate has a strong negative impact on the reproduction of burbot in Curonian Lagoon and the lower Nemunas River (Lithuania). Svagzdys related the decrease in catches with winter floods and thaws, which cause high mortality of eggs during their incubation.

**Figure 10.**
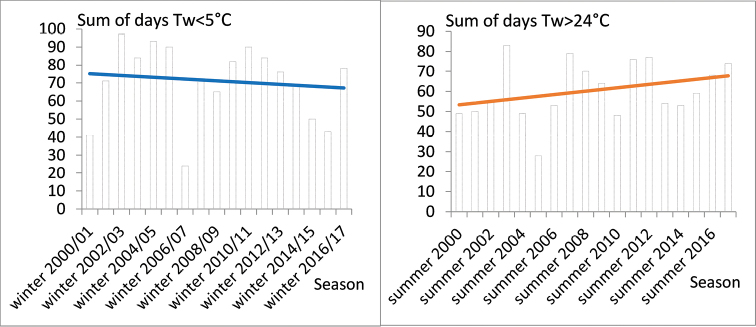
Sum of days with water temperature appropriate for reproduction and egg incubation (Tw <5 °C, left) and summer stress duration (Tw >24 °C, right) per seasons (data source: Executive agency for exploration and maintenance of the Danube river (EAEMDR)).

**Figure 11.**
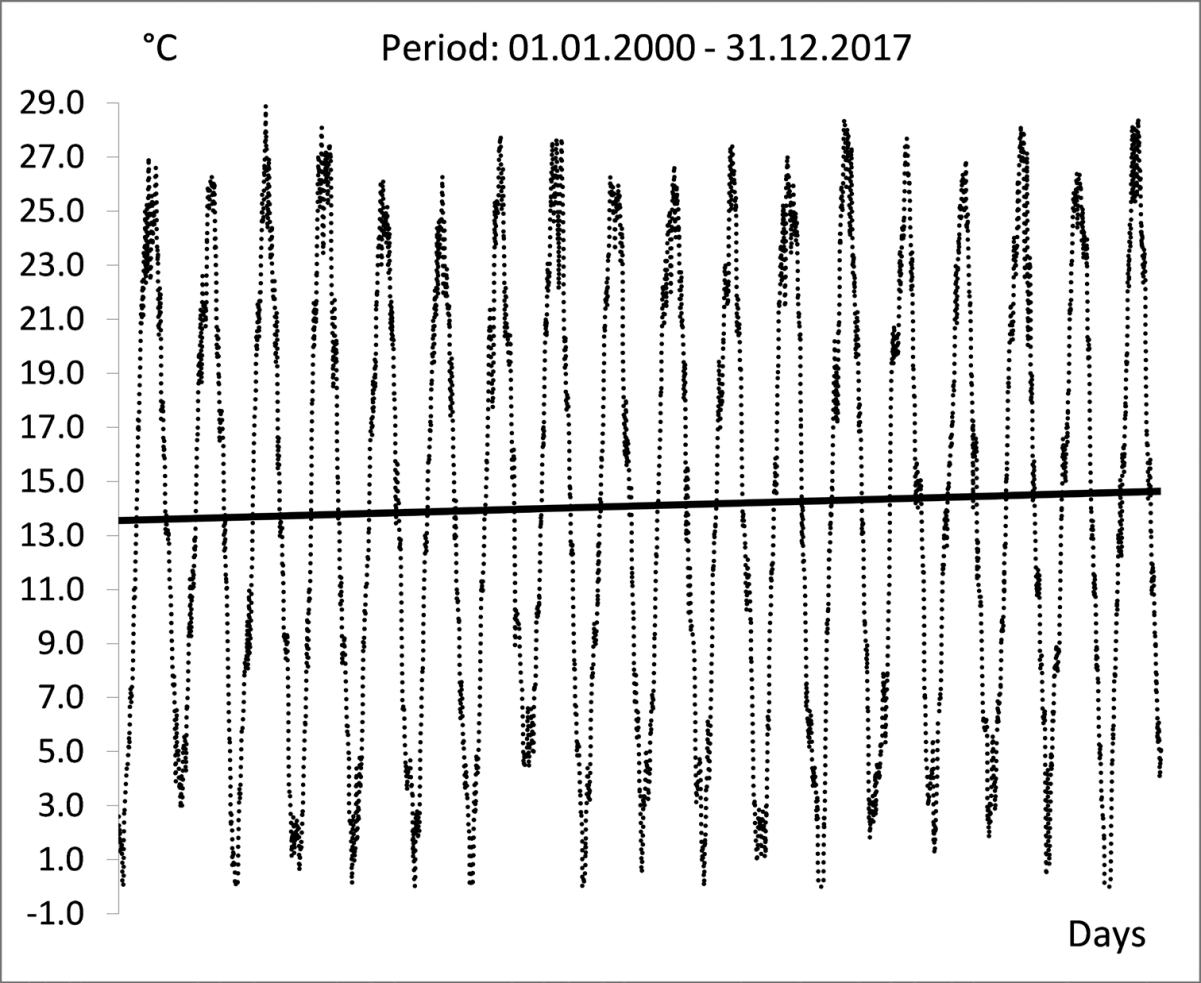
Daily water temperatures (°C) in Danube at Silistra between 01.01.2000 and 31.12.2017 (data source: Executive agency for exploration and maintenance of the Danube river (EAEMDR)).

Basarin et al. (2016) reported a statistically significant long-term warming trend in water temperature (Tw) at three sites (Bezdan, Bogojevo, and Veliko Gradište) along the Serbian Danube, both for annual and seasonal minimums and maximums, by analyzing the mean monthly Tw records from 1950 to 2012. Basarin et al. (2016) detected a general increasing in Tw beginning in the 1980s, as shown by a time series of annual mean values. A similar increase of the minimum and mean annual Tw of the Danube and its main tributaries in Croatia since 1988 was reported by Bonacci et al. (2008). A statistically significant increase (0.039 °C per year) in the mean annual Tw of the Danube at Bogojevo (Serbia) and increases in all the average monthly values between 1961 and 2013 were reported by Ducić et al. (2015). A comprehensive study on the trends in alterations of water temperatures across Elbe and Danube river basins warns that there is a significant risk for fundamental changes in river ecosystems, which may lead to significant distortions in community structure and composition Markovic et al. (2013). By analyzing long-term data sets, Markovic et al. (2013) found phase shifting in spring warming of almost two weeks, an increase in the number of the days with Tw above 25 °C, and an increase in the duration of summer heat stress. Although such studies are not available for the Lower Danube, data provided by EAEMDR from the hydrometric station at Silistra (km 375.5), confirms the amplification of the summer stress and shortening of the favorable reproduction period in the winter (Fig. 10). We calculated a decreasing trend in the number of the days with Tw below 5 °C during the winter (Fig. 10, left), a strongly increasing trend of prolongation of the summer stress with Tw higher than 24 °C (Fig. 10, right), and a trend of average increase of Tw with 0.056 °C per year (Fig. 11) for 2000–2017. The continuously warming water temperatures is disastrous for the future of Bulgarian burbot populations considering that incubation of burbot eggs in temperature above 5 °C causes high mortality (100% in the eyed-egg-stage) (Żarski et al. 2010) and summer stresses are prolonged.

Due to the observed dramatic decline over the last three generations and continuing negative influences, we recommend to increase the conservation status of this species in Bulgaria from Vunerable (Trichkova and Stefanov 2011) to Endangered (according to IUCN criteria CR A2cd, but down-listed to EN° after correction for regional level of assessment). In neighboring Serbia and Romania, where Burbot is much more widespread, it has the status of Least Concern and Vulnerable (Simonović 2001; Botnariuc and Tatole 2005; Oţel 2007). As a consequence of our research, we consider warming of water temperatures and fragmentation as the most important threats affecting the burbot in Bulgaria, and overfishing, pollution, and negative hydro-morphological changes as potential threats.
